# Neural classification of Norwegian radiology reports: using NLP to detect findings in CT-scans of children

**DOI:** 10.1186/s12911-021-01451-8

**Published:** 2021-03-04

**Authors:** Fredrik A. Dahl, Taraka Rama, Petter Hurlen, Pål H. Brekke, Haldor Husby, Tore Gundersen, Øystein Nytrø, Lilja Øvrelid

**Affiliations:** 1 Health Services Research Unit, Akershus University Hospital, Lørenskog, Norway; 2 Institute for Clinical Medicine, Campus Ahus, University of Oslo, Oslo, Norway; 3 Department of Linguistics, University of North Texas, Denton, TX USA; 4 Division of Diagnostics and Technology, Akershus University Hospital, Lørenskog, Norway; 5 Department of Cardiology, Oslo University Hospital Rikshospitalet, Oslo, Norway; 6 Data and Analytics, Akershus University Hospital, Lørenskog, Norway; 7 Department of Computer Science, Norwegian University of Science and Technology, Trondheim, Norway; 8 Department of Informatics, University of Oslo, Oslo, Norway

**Keywords:** Tomography, X-ray computed, Machine learning, Natural language processing, Reproducibility of results

## Abstract

**Background:**

With a motivation of quality assurance, machine learning techniques were trained to classify Norwegian radiology reports of paediatric CT examinations according to their description of abnormal findings.

**Methods:**

13.506 reports from CT-scans of children, 1000 reports from CT scan of adults and 1000 reports from X-ray examination of adults were classified as positive or negative by a radiologist, according to the presence of abnormal findings. Inter-rater reliability was evaluated by comparison with a clinician’s classifications of 500 reports. Test–retest reliability of the radiologist was performed on the same 500 reports. A convolutional neural network model (CNN), a bidirectional recurrent neural network model (bi-LSTM) and a support vector machine model (SVM) were trained on a random selection of the children’s data set. Models were evaluated on the remaining CT-children reports and the adult data sets.

**Results:**

Test–retest reliability: Cohen’s Kappa = 0.86 and F1 = 0.919. Inter-rater reliability: Kappa = 0.80 and F1 = 0.885. Model performances on the Children-CT data were as follows. CNN: (AUC = 0.981, F1 = 0.930), bi-LSTM: (AUC = 0.978, F1 = 0.927), SVM: (AUC = 0.975, F1 = 0.912). On the adult data sets, the models had AUC around 0.95 and F1 around 0.91.

**Conclusions:**

The models performed close to perfectly on its defined domain, and also performed convincingly on reports pertaining to a different patient group and a different modality. The models were deemed suitable for classifying radiology reports for future quality assurance purposes, where the fraction of the examinations with abnormal findings for different sub-groups of patients is a parameter of interest.

## Key points

- Neural models have been trained to classify Norwegian radiology reports from CT-scans of children, with high accuracy.
- The models performed well also on radiology reports from a different patient population (adults) and a different modality (X-ray images).
- The developed models are robust with respect to different contexts, and may be used in quality assurance processes.

## Background

Quality assurance (QA) of hospital radiology is an important field, and QA of routines for CT-scanning is of particular interest because it accounts for nearly 70% of the radiation exposure from diagnostic radiological procedures over-all [1]. Several large epidemiological studies performed world-wide in the past decade have identified increased risks of central nervous system tumours and other types of cancer related to diagnostic radiation exposure in childhood and adolescence [2–5]. Therefore, criteria for applying CT scans should be monitored in order to limit potentially harmful exposure. A basic QA parameter is the fraction of the examinations that produces positive findings for a given sub-group of patients, which may be a tool to indicate areas for review. In particular, it is interesting to identify possible subsets of patients that may have a rate of findings that might be too low to warrant the radiation risk, but also sub-groups where the rate is so high that it might indicate that the criteria used are too restrictive [6, 7].

The present article describes the development of a tool that can be used to estimate the fraction of positive findings in a set of radiology examinations for a hospital in Norway. The classification task is more complex than one might imagine, since the hospital’s written radiology reports are unstructured (or semi-structured), and lack a binary conclusion of positive findings. We examine the use of different machine learning (ML) techniques for the given natural language processing (NLP) task. According to the “AI Index” [8], which seeks to monitor the progress in artificial intelligence (AI) in a balanced way, ML with end-to-end training is currently the most successful approach for virtually all standardized NLP test problems. This refers to methodologies where the human experts only provide annotated or categorized text examples, as opposed to the inclusion of human knowledge engineering in the way of formalized syntax rules, ontologies or knowledge graphs. The present project follows this trend and uses end-to-end training with no human input except an expert’s labelling of positive and negative examples.

Within the field of clinical NLP [9], radiology reports have provided data for a number of systems performing tasks such as coding of findings [10], suggestions for repeat examinations [11] and detection of nosocomial infections [12]. The system presented in [13] automated the coding of radiology reports using the SNOMED CT reference terminology. Chapman et al. [14] present a document-level classification of CT pulmonary angiography reports using a standard Naive Bayes classifier. Whereas deep neural networks have been extensively employed to process medical images, there are still only a few studies that employ neural network architectures to process textual radiology reports. A recent study [15] makes use of a convolutional neural network to extract pulmonary embolism findings from thoracic computed tomography (CT) reports. Another study [16] employs a recurrent neural network to distinguish between fractures and non-fractures in a set of orthopaedic surgeon-classified reports.

The studies referred above were performed on English clinical text, and smaller languages are to a large extent under-studied within the field of clinical NLP, although this is slowly changing [17]. The present work provides the first published results for radiological text written in the Norwegian language. In addition to large differences in vocabulary, Norwegian also differs from English in having three genders for nouns and more inflection forms in general, as well as arbitrarily long compound words, much like German. This tends to produce a higher number of low-frequency tokens. Although Norwegian is placed in the German language family, it also has grammatical similarities with English, however, such as the tendency to end sentences with a preposition (although sometimes frowned upon in both languages), which is never seen in German. Our study is unique also in the use of an unselected sample of all CT-examinations of children in a general hospital over a substantial period of time, with the task of identifying reports that document any kind of abnormal findings across different medical specialties.

## Methods

The radiology reports under study are from Akershus University Hospital, which is located just outside Oslo. It serves a population of around 600.000 people and has close to 1000 beds. The primary data set consisted of all written reports from CT-scans of children (< 20 years) in the time period 2006–2017, for a total of 13.506. In addition, 1000 randomly chosen reports from CT-scans of adults and 1000 randomly chosen reports from chest x-ray of adults from the same hospital were included in order to test the models’ external validity.

The radiologist in the project (PH) labelled each report as negative or positive, according to his judgement regarding the presence of any description of abnormal findings, and we define this as the reference standard (RS). For all three data sets, abnormality was defined as any deviation from normal physiology, regardless of the clinical implications or the purpose of the given examination. The reports were semi-structured in the sense that most of them contained the token “R:” followed by summary (Children-CT: 85%, Adult-CT: 87%, Adult-X-ray: 47%). However, the summary seldom gave a binary conclusion that could be used for classification and frequently omitted details of abnormal findings that would be significant enough to trigger a positive classification.

In order to evaluate the reliability of the RS, a hospital clinician (PB) labelled a random sample of 500 children-CT reports. Prior to this, he discussed a different random sample of 100 children-CT reports with the radiologist, in order to align their evaluations. A clinician was chosen because he represents the users of the reports, and the agreement between those who write the reports and those who read them was deemed particularly important. In addition, the radiologist also re-labelled the same 500 reports after a period of three months as an intra-rater reliability test. The reliability tests were performed with Cohen’s kappa index. Statistical significance tests were performed with bootstrap, using 10.000 resampled data sets generated by random sampling with replacement [18].

**Fig. 1:**
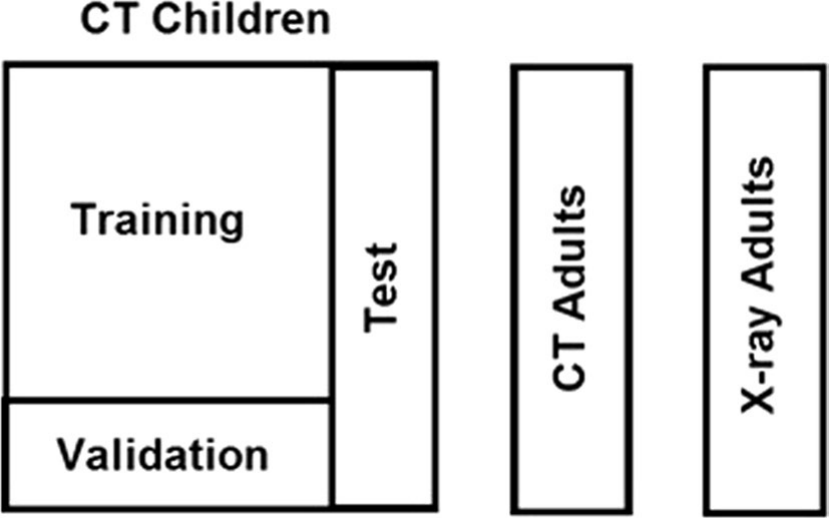
Data sets

Prior to any analysis, a test set consisting of 10% of the CT-Children reports was sampled at random, and stored together with the CT-Adults and X-ray Adults data sets. These data sets were not accessed until all model selection and training was finished, in order to guarantee unbiased performance estimates. The different ML algorithms had access to the remaining 90% of the CT-Children data, called the development data set. In order to eliminate overfitting, the development data set was split further into a training set (80%) and a validation set (20%), where the former was used to train a given model with a given setting of hyper-parameters, and the latter was used to evaluate the performance of the resulting model. This made it possible to search for hyper-parameters that give good performance on unseen data, thus avoiding overfitting. When suitable hyper-parameters had been determined, these were used to train the model on the full development data set. The models that were developed had different sets of hyper-parameters, and no formalized optimization method was used in the search. Rather, the researchers used their modelling experience and tested a broad selection of hyper-parameter configurations in an ad-hoc manner. The final models were evaluated on the held-out Test set, the CT-Adult set and the X-ray Adults set. Hence, all the performance estimates were created on data that were not a part of the model development or training. Figure 1 illustrates the various parts of the data set, where the development data set is the union of “Training” and “Validation”. The 500 reports used for reliability testing were sampled uniformly from the entire children-CT data set.

The chosen performance measures were AUC (area under the receiver-operator-characteristic curve) and F1-score. The AUC can be defined for models that give a numerical output and classify cases by comparing the output to a threshold, so that one can trade sensitivity for specificity by adjusting the threshold. The AUC has a convenient graphical representation that illustrates how the model’s sensitivity can be traded for its specificity. It can also be defined as the probability that a random positive case has a higher model score than a random negative one. The F1-score is commonly used in the ML and NLP communities. There are several alternatives to the F1 measure, including Cohen’s Kappa, which can all be computed from the confusion matrix in terms of True or False Positives (TP/FP) and True or False Negatives (TN/FN), as shown in Table 1. For completeness we report the TP/FP/FN/TN figures as well.

**Table 1:** The confusion matrix

	Actually positive	Actually negative
Classified as positive	True positives (TP)	False positives (FP)
Classified as negative	False negatives (FN)	True negatives (TN)

The F1 score is defined as the harmonic mean of the model’s positive predictive value (precision) TP/(TP + FP) and specificity (recall) TP/(TP + FN), and is given by the expression 2*TP/(2*TP + FP + FN) [19].

Although the main purpose of the clinician’s labelling (and the radiologist’s re-labelling) of the 500 children-CT reports was reliability testing, these were also evaluated by the F1-score. These scores were interpreted as reference values for human performance, against which the ML models were compared.

The radiology reports were extracted from the hospital’s EPR system DIPS and pre-processed using the UDPipe package, with the Norwegian model trained on the Norwegian UD treebank [20]. While this package supports more advanced pre-processing like syntax parsing, which could potentially be useful, the present application used only tokenization and sentence segmentation. Token in this context are essentially words (case insensitive) and punctuation marks that are not a part of standard abbreviations.

Although radiology reports to a varying degree mention body parts that were examined and found normal, the focus is on descriptions of abnormal findings. One would therefore expect to see an association between the report length and classification status. As a point of reference we therefore tested a trivial model that predicts the classification from the number of tokens in the report, only.

Our first nontrivial model was a support vector machine (SVM) over a bag-of-words representation of the texts. The model structure was linear, and term frequency–inverse document frequency (TFIDF) was used, which is a standard method for scaling the impact of words according to their frequency within and between documents [20]. No limit was set to the number of tokens in the selected configuration. SVM is arguably the most widely used non-neural ML method, which sometimes performs comparably to NN methods [21].

Subsequently, a bidirectional recurrent neural network (RNN) model of type long short-term model (LSTM) was developed [22]. LSTMs have a wide-spread use in NLP, mostly for sequence labeling tasks but also for text classification tasks such as the current task [23]. Simple feed-forward neural networks require that the input length has to be fixed, which often is not the case with sequential data. An LSTM model solves this by processing one input symbol at a time, and including the model’s own output from the previous step as additional input. In this way, the model has the ability to maintain information about previous symbols that have been read, and can be trained to produce a final output that summarizes the entire document (positive or negative, in the present application). The present application uses a configuration that combines an LSTM model that reads forward with one that reads backward, hence the label “bidirectional”. Vector representations of the input words were used, and these were randomly initialized and tuned during training. Different settings for hyper-parameters were explored, and the final model used 32 hidden units and word vectors of dimension 30. The token set was limited to the 10.000 most common ones. During training a 50% dropout rate was used.

A convolutional neural network (CNN) model was also developed, which solves the variable input length problem in a different way. It uses a kernel, in the form of a “sliding window” that is trained to identify features in short sub-strings of the input. The kernel output features are fed into a computational layer that filters out the maximum value for each feature (so-called “max pooling”). These are propagated through a hidden layer of neurons, which is connected to a single output node.

After hyper-parameter tuning, a model with a kernel width of 5, with 64 features and 10 hidden nodes was chosen, and the token set size was set to 5.000. Since Kim [24], CNNs have been widely used in NLP for text classification tasks, like sentiment analysis.

In order to illustrate the different models’ data requirements, we evaluated their F1-performances on the children-CT test set after training on sub-samples of varying size from the training data set. We also evaluated the F1-performances as a function of the number of tokens included for the models.

## Results

**Fig. 2:**
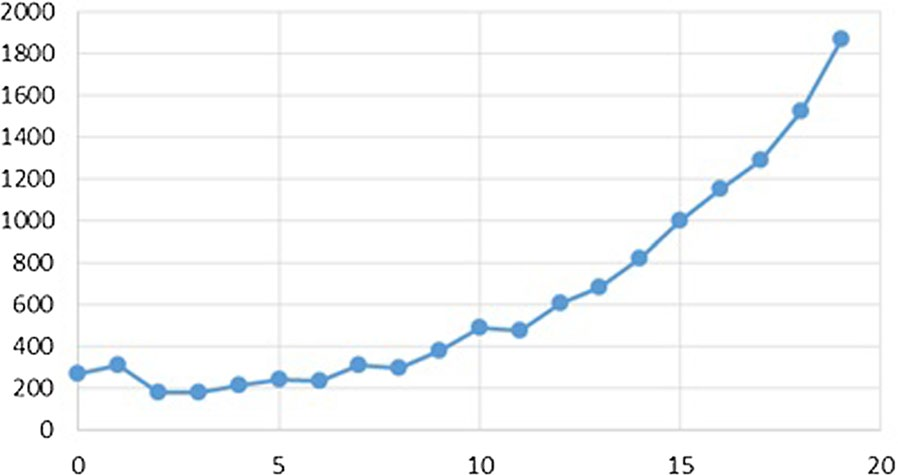
Age distribution of the CT children scan

We first give some descriptive statistics of the Children CT patients: 45% of the patients were female, while their age distribution is shown in Fig. 2. Rounded to full percentages, 26% were referred from the Emergency department, 24% from Paediatrics, 16% from Orthopaedics, 11% from Surgery, 6% from Internal medicine, 9% from external sources and 9% from other units, including Psychiatry. 68% were emergency cases.

All reports were written in Norwegian and the number of tokens per report varied from 3 to 402. The Children-CT, Adult-CT and Adult-X-ray data sets had an average of 61, 87 and 31 tokens per report, respectively. The development part of the Children-CT data set had a vocabulary of 22.033 unique tokens. This vocabulary covered 98% of the tokens in the test set reports, 95% of the tokens in the Adult-CT reports and 95% of the tokens in Adult-X-ray reports. (The cover of the respective vocabularies is lower, but this is irrelevant since tokens that are not seen during training are indistinguishable for the model.)

In the CT-children data set 45% of the reports were classified as positive. In the CT-adults data set 71% were positive and in the X-ray adult data set 66% were positive. There was a substantial uncertain area of reports that to a varying degree suggest possible findings without drawing firm conclusions, and in these cases professional judgement can go either way. An example of this is the following short report, translated from Norwegian: “*No enlarged intra-abdominal glands. The liver seems somewhat large, but the spleen is not enlarged. Unremarkable pancreas. No free intra-abdominal fluid.*” It is not immediately clear whether a liver that seems somewhat large represents an actual abnormality or not. Note also the typical abbreviated syntax with missing predicate verbs.

Table 2 gives the results of the human classifiers on the 500 random reports with Kappa-score, F1, precision (P = TP/(TP + FP)), recall (R = TP/(TP + FN) and the elements of the confusion matrix. Although the confidence intervals overlap substantially, the differences between the radiologist’s and clinician’s Kappa and F1-scores are statistically significant (p-values 0.019 and 0.014). This is because their misclassifications are strongly correlated.

**Table 2:** Reliability results

Tester	Kappa (CI)	F1 (CI)	P	R	TP/FP/FN/TN
Radioligist (re-test)	0.86 (0.82–0.91)	0.92 (0.90–0.94)	0.88	0.96	193/26/8/273
Clinician	0.80 (0.75–0.86)	0.89 (0.86–0.91)	0.86	0.92	184/31/8/17/268

The performances of the different ML models on the children-CT test set are given in Table 3. The F1-scores of the Bi-LSTM and CNN models are statistically significantly higher than the SVM F1-score (p-values 0.0142 and 0.0188). For comparison, the trivial model based only on word counts had an F1-score of 0.77 and an AUC of 0.86.

**Table 3:** Model classification results on the Children-CT test set

Model	Kappa (CI)	F1 (CI)	P	R	TP/FP/FN/TN
SVM	0.975 (0.967–0.983)	0.911 (0.894–0.929)	0.909	0.914	561/56/53/681
Bi-RNN	0.978 (0.970–0.986)	0.927 (0.913–0.941)	0.927	0.951	584/62/30/675
CNN	0.981 (0.974–0.988)	0.930 (0.917–0.944)	0.918	0.943	579/52/35/17/685

**Fig. 3:**
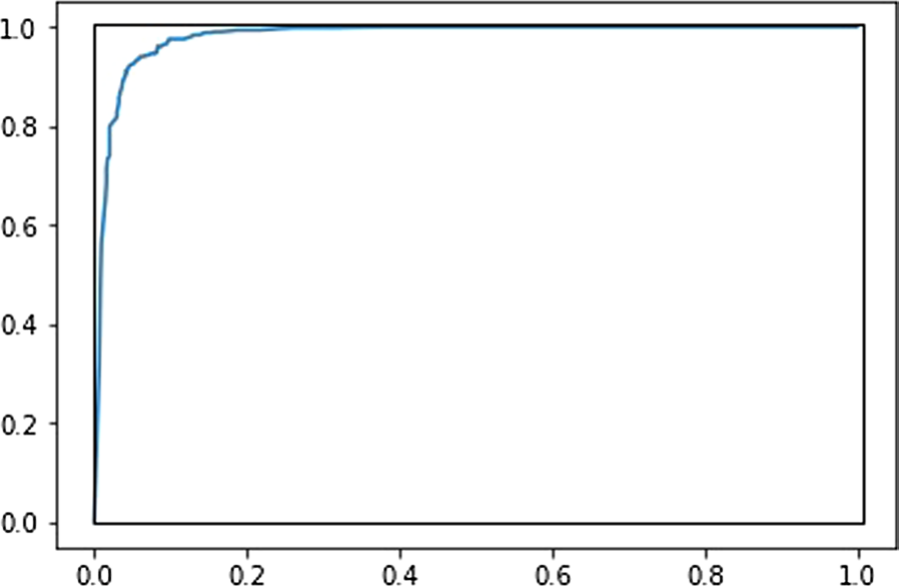
ROC-curve for the CNN classifier

Figure 3 shows the ROC-curve for the CNN classifier, where the area under the curve is 98.1% of the perfect performance, indicated by the rectangle. The ROC-curves for bi-LSTM and SVM are visually similar.

Tables 4 and 5 give the ML models’ performances on the adult-CT and adult-X-ray data sets, respectively.

**Table 4:** Model classification results on the adut-CT data set

Model	AUC (CI)	F1 (CI)	P	R	TP/FP/FN/TN
SVM	0.948 (0.934–0.962)	0.896 (0.877–0.915)	0.954	0.844	601/29/111/259
Bi-RNN	0.948 (0.934–0.962)	0.913 (0.895–0.930)	0.947	0.881	627/35/85/253
CNN	0.957 (0.944–0.970)	0.917 (0.900–0.934)	0.945	0.890	634/37/78/251

**Table 5:** Model classification results on the adut X-ray data set

Model	AUC (CI)	F1 (CI)	P	R	TP/FP/FN/TN
SVM	0.957 (0.944–0.969)	0.914 (0.897–0.932)	0.936	0.893	586/40/70/304
Bi-RNN	0.967 (0.956–0.978)	0.924 (0.907–0.941)	0.959	0.892	585/25/71/319
CNN	0.966 (0.955–0.977)	0.908 (0.890–0.926)	0.962	0.860	564/22/92/323

**Fig. 4:**
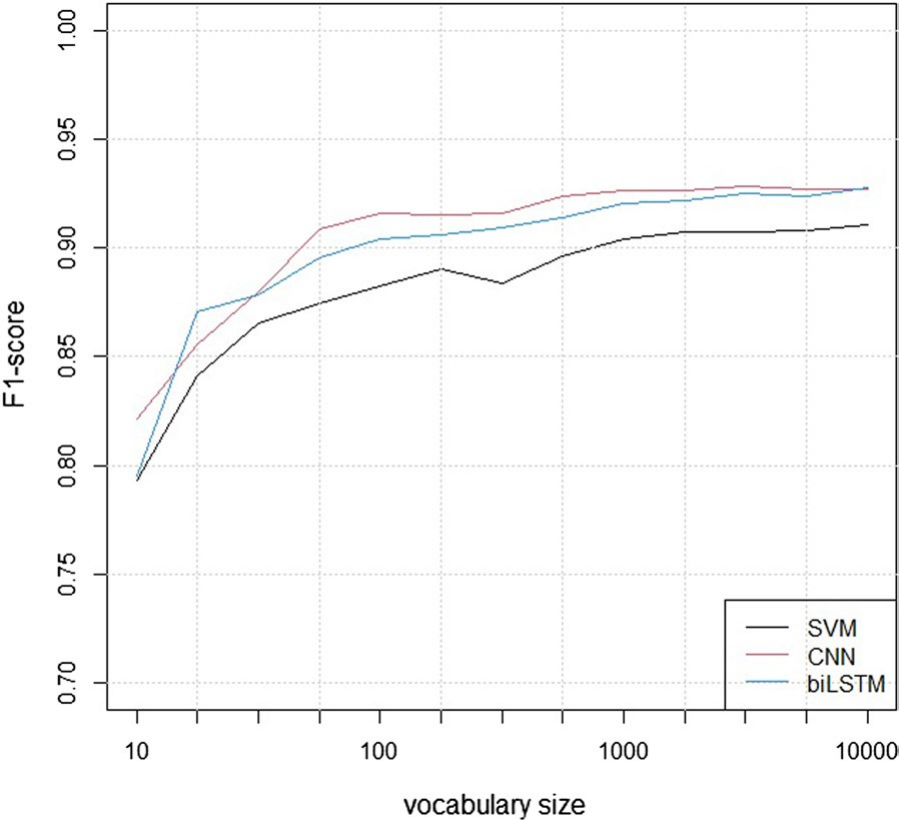
The F1-scores of the models for different vocabulary sizes

Figure 4 shows the analyses of the models’ sensitivity to the size of the vocabulary, using logarithmic X-axes. With a vocabulary of size 10, the strongest predictor for a negative classification for the SVM model was the word “ingen” (meaning “no”), while the strongest predictor for a positive classification was the word “med” (meaning “with”), closely followed by two other prepositions: “på” (meaning “on”) and “av” (meaning “of”).

**Fig. 5:**
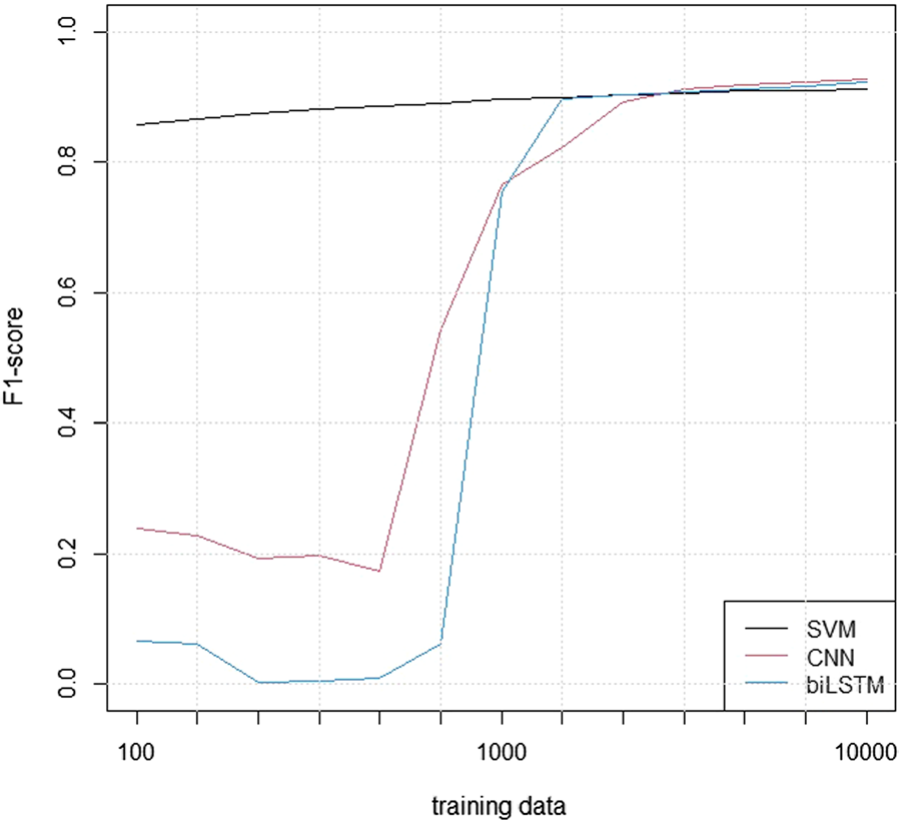
The F1-scores of the models for training set sizes

Figure 5 shows the analyses of the models’ sensitivity to the size of the training set, using logarithmic X-axes.

## Discussion

Our main finding is that all three models perform very well on the test data set, with AUC scores around 0.98 and F1-scores around 0.92. The F1-scores are clustered around the radiologist’s score, which may in practice represent an upper limit to the possible performance on this task. Although a report’s word count is associated with its classifications, this correlation was not strong enough to give reliable classifications by itself.

The study relied on the labelling effort of a single radiologist, which is a potential weakness. However, the reliability tests showed convincing results, with an inter-rater Cohen’s kappa level of 0.80 and a test–retest Cohen’s kappa of 0.86. A total of 15.506 reports were labelled, which is a very high number, and it is unavoidable that a human will make a few random errors. More importantly, there is a substantial uncertain area of reports that to a varying degree suggest possible findings without drawing firm conclusions, and in these cases professional judgement can go either way.

The neural models performed slightly better than SVM with higher F1-scores, which was statistically significant. The difference was mainly in the models’ specificity (recall). Over-all, the reports are written in a relatively simple (even over-simplified) syntax with few negations, which is probably beneficial for SVM relative to the neural models and may explain the small differences.

All three models showed robust performance as a function of vocabulary size, with reasonable performance for 10 tokens, adequate performance with 100 and close to maximum performance with 1000. It was unsurprising that the word for “no” was the strongest negative predictor in the smallest SVM model, but more of a surprise that the strongest positive ones were prepositions. The reason may be that descriptions of specific locations in the body were used mostly in the context of abnormal findings.

The analysis of training set sizes showed that the SVM model was relatively robust, while the performance of the neural models deteriorated quickly for training sets with fewer than 1000 reports. It is a well-known fact that neural models tend to require more data, but the present analysis is likely to overestimate this effect since the hyper-parameters were adjusted to the full data set.

The models were trained on a large and unselected material of CT-scan reports, spanning a wide range of medical specialities. Therefore, the models could not rely on simple disease-specific cues, and our goal was to create robust models that could generalize the concept of abnormal findings. This was confirmed, since the models performed well on the external validation data sets, with F1-score around 0.91 and AUC around 0.95. From a scientific point of view, it is very satisfying that these models can perform adequately outside of their original scope. For CT images of children and adults, the distribution of the underlying diseases will necessarily be different. One might therefore expect that a model trained from children reports could experience problems with reports that describe findings relating to diseases like heart attack or stroke that mostly affect the elderly. The favourable performance therefore indicates that the models do in fact capture a generic concept of abnormality. This conclusion is strengthened further by the fact that the models work well even on X-ray reports of adults, which are different in terms of modality as well as patient group. The goal of the study was to develop models that may be used for subsequent QA, and a robust performance is key for such applications.

The study of Chapman et al. [14] uses a syntax rule based text processing algorithm for identifying features in clinical report documents. Although not directly comparable to the present one, their most similar task appears to be disease classification, for which they reported an F1-score similar to ours (0.90). The present study has more similarities with that of Chen et al. [15], who also trained a CNN to classify radiology reports as positive or negative. They reported an AUC of 0.97 and an F1-score of 0.938, which are virtually identical to our results, so our study successfully replicates their results in a different setting. The languages are different, and an application of this kind to a Scandinavian language is novel. The other study used a larger set of radiology reports, but had fewer labelled training examples, so for the most relevant parameters the present study is larger. There are also methodological differences, as Chen et al. compared their CNN model to a pre-existing rule-based classifier, while we compared SVM, bi-LSTM and CNN models. The most important contribution of our study may be the successful validation of our models on radiology reports relating to a different population (adults versus children) and a different image modality (X-ray versus CT).

As there are major differences between the clinical importance of a positive or negative finding between different patient groups, it is not possible to define a specific level or range that is generally appropriate. However, knowledge of the current practice is essential in any QA work, and the rate of positive findings in a specific sub-group is a basic parameter of interest. We believe that our model may be applied in QA not only in our own hospital, but also others.

## Conclusion

Our study shows that SVM, CNN and bi-LSTM models can be trained to classify Norwegian radiology reports from CT-scans of children. While the neural models CNN and bi-LSTM perform slightly better, even SVM was sufficient for close to perfect performance. All the three models performed well also on CT-reports for adults and X-ray reports for adults. This shows that the models are robust with respect to different contexts and may be used in quality assurance processes.

Until the present, neural network language modelling has been a cutting-edge technology reserved for specialists in the field, but this is changing rapidly as the technology is maturing. In the near future, we expect applications like the present one to be as commonplace as today’s use of linear or logistic regression for quantitative analysis.

Our study also indicates that NLP tools can be used to chart our practice with a precision sufficient to initiate valuable discussions in our continuous effort to maintain good quality.

## Data Availability

Under Norwegian privacy law, the radiology texts cannot be made available because re-identification cannot be ruled out.

## Abbreviations

AI: Artificial intelligence
AUC: Area under the receiver operator characteristic curve
bi-LSTM: Bidirectional long short-term memory
bi-RNN: Bidirectional recurrent neural network
CNN: Convolutional neural network
CT: Computed tomography
HER: Electronic health record
ML: Machine learning
NLP: Natural language processing
RNN: Recurrent neural network
ROC: Receiver operator characteristic
SVM: Vector machine
